# Comparison of Efficiencies of Non-invasive Prenatal Testing, Karyotyping, and Chromosomal Micro-Array for Diagnosing Fetal Chromosomal Anomalies in the Second and Third Trimesters

**DOI:** 10.3389/fgene.2019.00069

**Published:** 2019-03-11

**Authors:** Yiyang Zhu, Qunda Shan, Jiayong Zheng, Qunxi Cai, Huanli Yang, Jianhong Zhang, Xiaodong Du, Fan Jin

**Affiliations:** ^1^Department of Reproductive Endocrinology, Women’s Hospital, School of Medicine Zhejiang University, Hangzhou, China; ^2^Department of Prenatal Diagnosis, Enze Women’s Hospital, Taizhou Hospital of Zhejiang Province, Zhejiang University, Taizhou, China; ^3^Taizhou Centers of Prenatal Screening, Taizhou Women and Children’s Hospital, Wenzhou Medical University, Taizhou, China; ^4^Department of Prenatal Diagnosis, Lishui Maternal and Child Health Care Hospital, Lishui, China; ^5^Department of Gynecology and Obstetrics, Wenzhou People’s Hospital, Wenzhou Maternal and Child Health Care Hospital, Wenzhou City Key Laboratory of Gynecology and Obstetrics, Wenzhou, China; ^6^Key Laboratory of Reproductive Genetics, Ministry of Education, Hangzhou, China

**Keywords:** non-invasive prenatal testing, chromosome microarray, karyotyping, copy number variant, prenatal diagnosis

## Abstract

In this study, we aimed to compare the efficiency of non-invasive prenatal testing (NIPT), karyotyping, and chromosomal micro-array (CMA) for the diagnosis of fetal chromosomal anomalies in the second and third trimesters. Pregnant women, who underwent amniocenteses for prenatal genetic diagnoses during their middle and late trimesters, were recruited at the Prenatal Diagnosis Center of Taizhou City. Maternal blood was separated for NIPT, and amniotic fluid cells were cultured for karyotyping and CMA. The diagnostic efficiency of NIPT for detecting fetal imbalanced anomalies was compared with karyotyping and CMA. A total of 69 fetal chromosomal imbalances were confirmed by CMA, 37 were diagnosed by NIPT and 35 were found by karyotyping. The sensitivities of NIPT and karyotyping for diagnosing aneuploidy were 96.3% and 100% respectively. Only one mosaic sexual chromosome monosomy was misdiagnosed by NIPT, whereas the sensitivity of NIPT and karyotyping was 70% and 30%, respectively, for detecting pathogenic deletions and duplications sized from 5–20 Mb. Taken together, our results suggest that the efficiency of NIPT was similar to the formula karyotyping for detecting chromosome imbalance in the second and third trimesters.

## Introduction

The prevalence of chromosomal anomalies declines following development of the fetus. Initially, the incidence of aneuploidy in the human embryo is more than double in women with a mean age of 38 years (Kuliev et al., 2005; Gianaroli et al., 2010). Most aneuploid embryos would undergo apoptosis or be spontaneously aborted after implantation. Additionally, the incidence of aneuploidy is less than 10% at early gestational period and further declines to 1% after the second trimester (Maxwell et al., 2016). However, the structural variations of chromosomes, such as deletions and duplications, which although non-lethal are usually associated with developmental and mental syndromes, would not be reduced following the gestational weeks (Capalbo et al., 2017). Moreover, the incidence of submicroscopic chromosomal anomalies even exceeded aneuploidy in the fetuses with ultrasonically detected abnormalities at the third trimester (American College of Obstetricians and Gynecologists Committee on Genetics, 2013). Thus, high resolution chromosomal testing has gradually become routine since 2012, with the chromosome microarray (Wapner et al., 2012) replacing karyotyping as the “gold standard.” Compared to karyotyping, CMA has a significantly shorter laboratory turnaround time and lower failure rate, which is critical for patients with indications of anomalies in ultrasound results (Petersen et al., 2017). As for those women with abnormal serum screening results and advanced maternal age, complete replacement of the traditional karyotyping method by CMA is controversial (Hay et al., 2018), considering both the financial burden as well as the difficulty in detecting balanced structural abnormalities and mosaics.

Another practical approach for detection of chromosome anomalies is NIPT (Warsof et al., 2015). The rapidly evolving next-generation sequencing (NGS) technique brings a revolutionary change in prenatal diagnosis, which makes whole-genome sequencing much faster and more economically feasible. By comparing the copy number variants, NGS has shown accuracy comparable to CMA and has been applied in preimplantation genetic screening for chromosome mapping to reduce the costs (Jin et al., 2015). NIPT, a milestone in NGS technology, provides excellent accuracy for fetal diagnosis of trisomy 21 and other aneuploidies when applied for the detection of cell-free fetal DNA (cffDNA) from maternal blood.

However, cffDNA originates primarily from the apoptosis of the placental syncytiotrophoblast and does not represent the complete fetal genome, particularly in cases of placental mosaicism (CPM) (Mardy and Wapner, 2016). False positive/negative results could occur because of inefficient sequencing depth or low fetal DNA template yields (Yin et al., 2015; Xu et al., 2016). Although NIPT has been applied to detect fetal chromosomal duplication/deletion syndrome, the overall efficiency of NIPT compared to traditional karyotyping or CMA has not been elucidated (Benn, 2016; Beulen et al., 2016; Oneda et al., 2016; Rose et al., 2016). Furthermore, cases of women with diagnoses of fetal structural abnormality but normal NIPT results at early gestational weeks, requiring additional fetal karyotyping and/or CMA testing, remain debatable.

Currently, NIPT applied in clinical tests for detecting aneuploidy is a low-coverage whole-genome sequencing technique that is theoretically capable of detecting 5 Mb CNVs with a sequencing depth of 3.5 Mb useable reads (Liu et al., 2016). This resolution size is similar to the standard 350-band karyotyping (Trask, 2002). In the present study, a prospective self-controlled diagnostic trial was carried out to evaluate the efficiency of the NIPT, karyotyping and CMA for detection of fetal chromosomal abnormality.

## Materials and Methods

### Design and Population

The clinical study participants were recruited from the Prenatal Clinic of Taizhou Hospital, Zhejiang, China, between January 2016 and July 2017. Women with single pregnancy who needed middle or late trimester amniocenteses for CMA testing with indications of fetal ultra-sonographic structural anomalies or positive maternal serum biochemical screening were recruited. Formula amniocenteses were performed before the 24th gestational week for indications of serum screening, for those considered risky or aging over than 35 years old. Because China’s population policy permits couples to obtain an abortion after the 28th gestational week if the fetus exhibits a severe malformation or genetic disorder, a third trimester invasive prenatal diagnoses should be performed if additional ultrasound structural anomalies were detected. Women with multiple pregnancies, a history of abortion risks, acute infectious disease or allergenic blood cell transfusion were excluded. The recruitment process is elaborated in detail in the Supplementary Material: flow chart 1; in total 813 participants were recruited and 802 of them received the available results. The indications of the invasive procedure are presented in Table 1; 609 cases with a sole indication and 193 combined with other indications. The study was approved by the Ethics Committee for Reproductive Medicine of Taizhou Hospital (ClinicalTrials.gov ID: NCT03201666).

**Table 1:** Demographic characteristic of samples.

Variables	Mean/cases
Cases of participants	802
Median age (years)	31 (17–46)
Median gestational weeks	23 (16–38)
Indications for amniocenteses	
Ultrasound abnormal	324 (40.1%)
Age ≥ 35 years	213 (26.4%)
Positive biochemical screening	449 (55.6%)
History of birth defect	46 (5.7%)
Parameters for NIPT	
useable Reads(Mb)	3.97 ± 0.62
Average cffDNA concentration(%)	13.30 ± 5.65
Chromosomal anomalies	
Total imbalance(confirmed by CMA)	69 (8.5%)
Aneuploidy	27 (3.3%)
Deletions/duplications	42 (5.3%)
Pathogenic or likely pathogenic del/dup^∗^	29 (3.6%)


*^∗^ Deletions/duplications were categorized as pathogenic, likely pathogenic, uncertain significance, or benign. CNVs inherited from normal phenotype parents were regarded as benign, and new fetal mutations were regarded as likely pathogenic.*

Before invasive procedures, participants signed a written informed consent form stating the risks of amniocenteses and the possibility of further detection for uncertain clinical significance of CMA/NIPT. Maternal peripheral blood (10 ml) was drawn in tubes containing EDTA prior to amniocenteses. Invasive procedures were performed using 22-gage needles under ultrasound guidance and 30 ml amniotic fluids were aspirated. For each sample, the CMA and culture cell karyotyping were carried out in parallel. The CMA was performed at the Hangzhou Jin Yu Lab, and Karyotyping and NIPT were analyzed at the Cytogenetic and Molecular Laboratory of Taizhou Hospital, respectively. The technicians analyzed the results in a blinded manner.

### NIPT

The NIPT procedure was performed using the Ion Proton Sequencer (Life Technologies) in a semiconductor platform at 400 flows according the document reported (Yin et al., 2015). Maternal blood plasma was separated by centrifugation at 1,600 × g for 10 min at 4°C, and cell-free DNA was extracted using the QIAamp DSP DNA Blood Mini Kit (Qiagen). The DNA products were stored at 80°C before commencing library construction. For library construction, plus Fragment Library Kit V3, Ion plus Fragment Library Adapters Kit (Life Technologies, United States) and AMPure XP beads were used following the manufacturer’s instructions. DNA libraries were quantified with Qubit12.0. There were 12 libraries consisting of 100 pM per sample, mixed and enriched by Ion One Touch^TM^ 2 Instrument (Life Technologies, United States). The templates then loaded onto one Ion PI^TM^ Chip v2 and sequenced using the Ion P1 Hi-Q200 V3 Kit (Life Technologies, United States) on the Ion Proton sequencing platform. We controlled the sequencing depth at 3.5 Mb and the usable reads at less than 2.5 Million or Z absolute value from 1.96 to 3 would re-construct the library. This sequence depth is theoretically capable of detecting 5 Mb CNVs with 3.5 Mb usable reads (Li et al., 2016).

### Karyotyping

The amniotic fluid cells were cultured following the local prenatal diagnostic standards. After centrifugation, the amniotic fluid cell suspension was inoculated in 50 ml bottles with Ham’s F10 (Hangzhou Biosan, Inc.). The culture medium was changed at day 6, and cells were harvested when more than 15 cell colonies appeared in the culture. For late gestational weeks, the medium was changed again, and the culture was prolonged if the number of cell colonies was insufficient for harvest after day 12. After colchicine treatment, digestion, harvest, hypotension, fixation, spread preparation and banding, karyotyping was carried out sequentially by standard chromosome analysis at 350-band level with ISCN 2016 (McGowan-Jordan et al., 2016).

### CMA

Affymetrix CytoScan 750 K chips were used for CMA analysis following the CytoScan^TM^ Assay Manual Protocol (Affymetrix, 2015). The chip contains 75000 probes that cover all International Standard Cytogenomic Array (ISCA) CNVs and can discern micro deletion/duplication with size >100 Kb. Amniotic fluid fetal DNA was extracted by Qiagen kit and 10 ng of amniotic fluid DNA was compared to maternal blood DNA by STR-PCR to rule out contamination if red blood cells were found in the precipitate. The DNA was processed by sequential steps of digestion, PCR, PCR-product check, purification, quantization, fragmentation, and QC gel labeling, hybridization, washing, staining, and scanning following the manufacturer’s protocol. The data were analyzed using Affymetrix Chromosome Analysis Suite Software (version3.1.0.15) (r9069).

For chromosome anomalies detected in this study, only imbalance rearrangements (aneuploidy, structural deletion and duplication) were considered during statistical analysis and balanced translocation, inversion, and loss of heterozygosity were excluded. The Database of Genome Variants^1^, UCSC genome browser^2^, ISCA Consortium web site ^3^, and DECIPHER (Database of Chromosomal Imbalances and Phenotypes using ENSEMBL Resources^4^) were used in the study to interpret our results. CNVs were categorized as pathogenic, likely pathogenic, of uncertain significance, or benign based on the region, size, gene content, and inheritance pattern (Capalbo et al., 2017). All CNVs of uncertain significance were compared to the parents’ genome; CNVs inherited from normal phenotype parents were regarded as benign, whereas new fetal mutations were regarded as likely pathogenic.

### Statistic and Sample Size Calculator

Data processing and statistical calculations were performed by Microsoft Excel and SPSS 22, respectively. Prenatal diagnostic efficiency, including sensitivity (Sen) and specificity (Spe), was defined as the ratio between either true positives or true negative cases of NIPT or karyotyping in CMA results, respectively. The positive predictive value (PPV) and negative predictive value (NPV) were defined as the ratio between either true positive or true negative cases of NIPT and karyotyping results, respectively. Since only pathogenic or pathogen-like CNVs were involved in the prognosis and clinical decisions, the benign CNVs were treated as negative results for efficiency calculation purposes. A receiver operating characteristic (ROC) curve, which was created by plotting the sensitivity and 1-specificity, was used to illustrate the diagnostic ability of NIPT and karyotyping at various CNV sizes. The area under the curve (AUC) of both techniques was measured and the cutoff points of resolutions of NIPT and karyotyping for diagnosing the low limit size of CNVs were determined by curves. The previously published reports (Yin et al., 2015; Liu et al., 2016) showed expected sensitivity towards NIPT for detecting CNVs over 1 M compared to the 100 Kb CMA to be 80% and specificity 90%, whereas for karyotyping this rate was 79.6% and 99% (Wapner et al., 2012), respectively. The sample size was calculated by software according to the above rates and a usable sample size of 61 patients was required in the anomalies group, with an allowable error of 0.1 in a two-tailed test with *p* < 0.05.

## Results

Between January 2016 and July 2017, 829 of 1053 pregnant women with indications for CMA undergoing invasive procedures were accepted for the trial. Sixteen were excluded because they had undergone multiple pregnancies. In all, 813 women were accepted both for the invasive procedures and NIPT testing. Of these, 11 in total were excluded because testing failure (3 for NIPT, 2 for CMA, other 6 for karyotype). However, all amniotic samples were successfully cultured before 32 gestational weeks. Thus, we obtained 802 cases for complete analysis. Five cases were found to have more than 2 anomalies that were calculated. The demographic characteristics are elaborated in detail in Table 1.

We confirmed 69 imbalanced chromosomal anomalies, with 27 instances of aneuploidy (Table 2). Thirteen of 42 deletions/duplications were categorized to be benign or inherited from normal phenotype parents and were not included for efficiency calculating. The accuracy and efficiency of NIPT and karyotype compared to CMA are presented in Table 3. NIPT showed similar accuracy, sensitivity and specificity as the karyotyping. We found 1 case of sex chromosome monosomy that was misdiagnosed by the NIPT technique and was confirmed to be a mosaic 45, X [48]/46, XX [62] both by the karyotyping and CMA methods. However, NIPT and CMA detected each of the other 3 true mosaics found by karyotyping: 47,XY,+12[10]/46,XY[70], 47,XY,+18[38]/46,XY[51], 45,X[13]/46,XX[57], whereas karyotyping misdiagnosed another 30 Mb duplication (16p13.1-11.2), with a mosaic percentage of 15% confirmed by CMA.

**Table 2:** Category of aneuploids detected by CMA, NIPT, and karyotype.

Aneuploids	NIPT(+)	Karyotype (+)
arr(9)^∗^3	1	1
arr(12)^∗^2–3	1	1
arr(13)^∗^3	1	1
arr(18)^∗^2–3	1	1
arr(18)^∗^3	6	6
arr(21)^∗^3	10	10
arr(X)^∗^1	3	3
arr(X)^∗^1–2	1^#^	2
arr (X)^∗^2,(Y)^∗^1	2	2
sum	26	27


*^#^ One case of mosaic 45X/46XX being misdiagnosed by the NIPT technique that was confirmed both by the karyotyping and CMA methods.*

**Table 3:** List of pathogenic CNVs with sizes lager than 2 Mb.

pathogenic or likely dup/del confirmed by CMA	NIPT	karyotype	CNVs size	unbalanced type
18p11.1(13,289,942-20,619,752)^∗^3	-	+	2.5	dup
5p15.33p14.3(113,576-18,961,778)^∗^1	+	+	18.8	del
4p16.3-15.33(3,890,466 - 17,492,847)^∗^1	+	+	13.7	del
13q33.1q34(102,381,998-115,107,733)^∗^1	+	-	12.7	del
5q35.1q35.3(172,795,779-180,715,096)^∗^1	+	+	7.9	del
22q11.21(18,631,364-21,800,471)^∗^1	-	-	3.2	del
17q23.1q23.2(58,097,017-60,339,091)^∗^3	+	-	2.2	dup
14q21.3(50,729,861-50,821,428)^∗^1	-	-	9.3	del
Xp21.3p21.1(28,808,531-35,822,212)^∗^1	-	-	7.0	del
Xq27.1q28(138,487.888-154,930,046)^∗^2	-	-	16.4	dup
2q13-14.1(108,730,140 - 119,858,077)^∗^3	+	-	11.0	dup
16p13.1-11.2(203,350 - 30,420,433)^∗^2-3	+	+	30.0	dup
dup1q32.1-q42(202,164,173 - 229,936,143)^∗^3	+	+	27.0	imbalanced translocation^#^
xq24-q28(120,715,800 - 156,040,894)^∗^1	+	+	35.0	imbalanced translocation^#^


^#^ Variants were imbalanced structural rearrangements which sourced from translocation parents.

**FIGURE 1:**
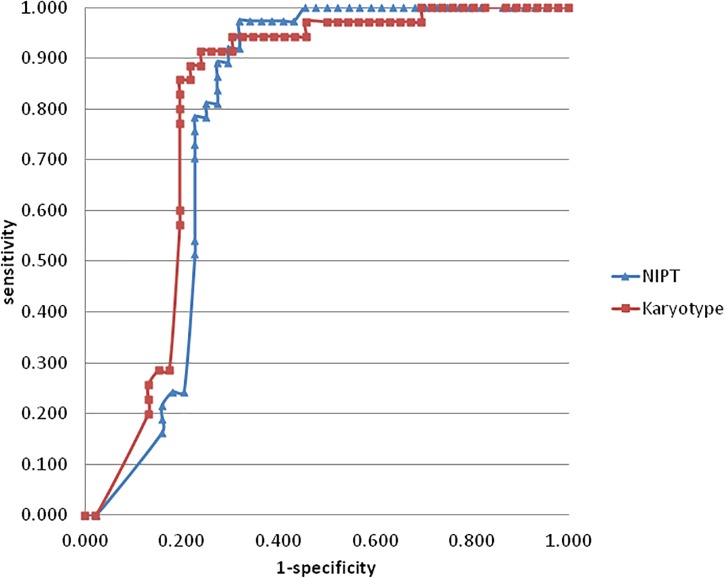
The ROC curve was created by plotting the sensitivity (horizontal axis) and 1-specificity (horizontal axis) of NIPT (blue) and karyotyping (red) for chromosomal imbalance var from 0.1–160 Mb. the AUC of NIPT and karyotyping were 0.786 and 0.81, respectively (*Z* = 0.314, *p* = 0.363). The curve cutoff was calculated by formula sensitivity + specificity-1, the maximum values of NIPT and karyotyping were 0.655 and 0.675, which corresponded to the resolution values of 5.4 M and 13.2 M, respectively.

As for chromosomal structural imbalance, 15 pathogenic CNVs sized from 0.063 to 1.6 Mb were detected by CMA only; neither NIPT nor karyotyping could detect CNVs with sizes smaller than 2 Mb (Supplementary Table S1). There were 14 pathogenic CNVs ranging from 2.2 to 35 Mb which were confirmed by CMA in this study, 9 of which were detected by NIPT whereas karyotype detected only 7 (Table 4). Most of these pathological variants were solely del/dup; only two variants were imbalanced structural rearrangements, which sourced from translocation parents. Interestingly, NIPT presented a slightly higher sensitivity than karyotyping for detecting CNVs with size ranging from 5 to 20 M (Table 5). The ROC curve comparing the resolutions of NIPT and karyotyping for chromosomal imbalance is shown in Figure 1. The CNV sizes varied from 0.1–160 Mb. The AUC of the two methods was also similar (*Z* = 0.314, *p* = 0.363), whereas the resolution cutoff of NIPT for detecting CNVs was 5.4 Mb (sensitivity = 0.973, specificity = 0.682), and this cutoff was 13.2 Mb for karyotyping (sensitivity = 0.914, specificity = 0.761). These results suggested that although NIPT presents a lower PPV, it has a similar sensitivity compared to traditional prenatal diagnosis techniques.

**Table 4:** Efficiency of NIPT and karyotype compared to CMA for detecting chromosome imbalance.

	Sensitivity	SE	Specificity	SE	PPV	SE	NPV	SE
**Efficiency for detecting total anomalies(imbalance)**								
karyotype	0.507	0.060	0.993	0.003	0.875	0.052	0.956	0.007
NIPT	0.536	0.060	0.984	0.005	0.755	0.061	0.958	0.007
**Efficiency for detecting aneuploidy**								
karyotype	1.000	0.000	0.996	0.002	0.900	0.055	1.000	0
NIPT	0.963	0.036	0.993	0.003	0.839	0.066	0.999	0.001
**Efficiency for detecting del/dup (pathogenic or likely pathogenic)**								
karyotype	0.241	0.079	0.995	0.003	0.636	0.145	0.971	0.006
NIPT	0.310	0.086	0.996	0.002	0.750	0.125	0.973	0.006


*^∗^ Sensitivity and specificity (Spe), was defined as the ratio between either true positives or true negative cases of NIPT or karyotyping in CMA results, respectively. The positive predictive value (PPV) and negative predictive value (NPV) were defined as the ratio between either true positive or true negative cases of NIPT and karyotyping results, respectively. No significant difference was found between the two methods.*

**Table 5:** Efficiency of NIPT and karyotyping for detecting pathogenic CNVs at various resolution levels.

CNV Size	NIPT				Karyotyping			
	Sen	Spe	PPV	NPV	Sen	Spe	PPV	NPV
>20M	0.967	0.988^∗^	0.763^∗^	0.999	1.000	0.997^∗^	0.938	1.000
5–20M	0.700^∗^	0.999	0.875	0.996	0.300^∗^	0.999	0.750	0.990
1–5M	0.100	0.999	0.500	0.988	0.100	1.000	1.000	0.988
<1M	0.000	1.000	0.000	0.985	0.000	1.000	0.000	0.985


^∗^The rates between the NIPT and karyotyping were significantly different (*p* < 0.05).

Additionally, in total 13 balanced structural rearrangements were detected only by karyotyping, six of nine inversions were variant inv (9) (p12q13), while the other three were inv (1) (p12q12), inv (19) (p12q11), and inv(Y)(p11q11). One complex translocation including t(2;12)(q36;q13.11), t(3;6)(q13.33;q21), t(9;10)(q11.1;p11.1) had been confirmed as a *de novo* case, while three other balanced translocations: t(1;12) (q12;q13.13), t(4;19) (q31.3;p12), t(5;9) (p15.3;p22), were inherited from one of the parents.

## Discussion

The prospective study described in this manuscript compared the efficiency of NIPT and karyotyping with CMA (100 Kb resolutions). The standard NIPT method showed similar accuracy levels when compared to karyotyping for the detection of chromosomal imbalances for both aneuploidy and duplications/deletions. Furthermore, we verified that the theoretical cutoff point of NIPT with 3.5 Mb sequencing reads for diagnosing micro-imbalance is 5 Mb (5.4 Mb in this study). Although it may present some false positive detections, the standard NIPT could still be utilized for screening chromosome duplications/deletions in the second and third trimesters.

### Expanding Indications for NIPT

Initially, indications for NIPT replaced the serum biochemical screening method for detecting aneuploidy in early or middle pregnancy stages (Warsof et al., 2015). Whether this technique is useful in the late trimester stage is disputed because chromosomal anomalies during this period are mainly characterized by structural imbalances (Wapner et al., 2012; American College of Obstetricians and Gynecologists Committee on Genetics, 2013). Mechanistically speaking, the potential uses of whole-genome NIPT sequencing of maternal plasma fetal DNA is not limited only to the detection of aneuploidy. Theoretically, even in low-coverage whole-genome sequencing studies, NIPT can still detect CNVs sized smaller than 5 Mb (Yin et al., 2015; Li et al., 2016). In this study, NIPT has the capability to detect 2.2 Mb micro-deletions and confirmed 64% CNV with a size from 2–20 Mb. From this aspect, the present 3.5 Mb usable reads sequencing depth is enough for detecting aneuploidy and deletion/duplication compared with 350-band karyotyping.

However, NIPT failed to detect any chromosomal imbalances with size <2 Mb, which may reflect its limitation to deep-sequence or reading low-concentration maternal blood cff-DNA templates (Benn and Cuckle, 2014). Low-coverage whole-genome sequencing was sufficient for detecting chromosomal imbalances as small as 0.1 Mb in amniotic samples (purely fetal DNA). In the present study, the average cffDNA concentration of samples was 13% (12% and 14% in second and third trimester, respectively), with a significant linear relation to the number of gestational weeks (*B* = 0.27, *p* < 0.001).

Although, our samples may have had some selection bias based on abnormal ultrasound indications for third trimester amniocenteses, subgroup analysis showed that the sensitivity of NIPT detecting duplication and deletions increased from 12.5% to 44.4% (*p* = 0.024) following longer gestation, and this increase was not significant in the culture karyotyping group (*p* = 0.351). Thus, our clinical data analysis confirmed the results from the Yin laboratory (Yin et al., 2015) that suggested that the accuracy increases with the increase of fetal DNA concentrations. However, due to sample size limitations in the present study, we failed to draw a concentration-dependent curve.

### Translocation and Mosaic

Although NIPT is a safe and economically sustainable technique, it suffers from limitations characteristic of all molecular whole-genome mapping techniques to detect balanced rearrangements compared to karyotyping. Nine inversions have been detected by karyotyping in the present study and most of those inversions were regarded as benign polymorphisms. Only four translocations have been detected in our study and three of them were inherited from parents carrying these translocations. Another limitation of NIPT is that fetal fraction DNA originated from syncytiotrophoblast apoptosis, which results in unavoidable false positives/negatives caused by placenta mosaic. General placenta mosaics are caused by mitotic non-disjunction events in the early stages of cell division or mitotic correction following a meiotic error (rescue) (Mardy and Wapner, 2016), and the prevalence of placenta mosaic in human embryo is unclear. Early mammalian embryo research findings suggested that trophoblasts differentiate from several vegetal poles simultaneously, which may give rise to more mosaic incidences than in the inner cell (Plachta et al., 2011; Ajduk and Zernicka-Goetz, 2013). In the present study, five mosaic cases were detected by chromosomal analysis and three of them were confirmed as true mosaics by CMA and repeated karyotyping after cordocenteses. One mosaic X monosomy was misdiagnosed by NIPT. On the other hand, one mosaic deletion was detected by NIPT and CMA but misdiagnosed by karyotyping, which means the genome mapping techniques are capable of detecting mosaics to a certain extent. Thus, high-throughput sequencing seems adequate for diagnosing chromosomal imbalances in late pregnancy.

### What Is the Choice in the Later Trimester?

Although suggestions to expand the chromosome examination to the micro- duplication/deletion level in the clinic are disputed (Capalbo et al., 2017), the latest consensus is that fetuses with the ultrasound anomaly should be subjected to clinical detection techniques beyond standard karyotyping (American College of Obstetricians and Gynecologists Committee on Genetics, 2013; Beulen et al., 2016; Oneda et al., 2016). It was reported that CMA can diagnose an additional 1.7–6% pathogenic or likely pathogenic chromosome anomalies compared to culture karyotyping (McGowan-Jordan et al., 2016) following high-risk indication from ultrasound anomaly screening procedures. This was also confirmed by our study, with a total of 2.8% of pathogenic CNVs sized >100 Kb being additionally detected in karyotyping negative samples. From this perspective, the invasive procedures should not be limited in late pregnancy stages, particularly for those with ultrasound abnormalities. Furthermore, fetal miscarriage associated with amniocenteses might decline as gestational weeks increase (Gordon et al., 2002; Oneda et al., 2016). As for karyotyping, although amniotic fluid cell culture has a sufficient detection success before 32 gestational weeks, the procedure requires at least a 2-week culture cycle. This additional *in-vitro* culture period could also increase the chances of false mosaics. Thus, the advantage of using amniotic fluid cells for karyotyping is avoiding the more invasive procedure, the cordocentesis, in later pregnancy stages. In addition, amniotic fluid cells are more suitable for the evaluation of mosaic forms of sex chromosomes compared to the fetal blood because of its embryonic origin. This has become less important in the era of molecular genome mapping, as both high-throughput sequencing and CMA chip techniques can replace the cordocenteses and provide a short laboratory turn around (usually in two days), which meets the urgent detection requirements of late-pregnancy diagnoses. According to our experience, offering NIPT is sufficient in patients who have a moderate risk of having a chromosomally abnormal fetus due to the presence of ultrasonographic soft marker(s) during last trimester of pregnancy. For those patients having indications based on an appearance of severe fetal malformation(s), especially combined with fetal growth restriction, CMA on native amniotic fluid cells should be performed.

## Conclusion

Our study may have a selection-bias drawback caused by non-randomized recruiting and those indications of ultrasound findings in the study participants increased from 28.4% to 61.8% between second to third trimesters. A larger sample size for future prospective studies is necessary to get better statistical reliability. Even with such limitations, we were able to show that NIPT sequencing with low-coverage whole-genome library sequencing displays similar accuracy levels compared to the 350-band karyotyping method for detection of chromosomal anomalies. We suggest that NIPT might be more sensitive to micro-duplication/deletion events, which can be confirmed downstream by more precise techniques such as CMA or CNV sequencing to verify the positive results.

## Data Availability

The raw data supporting the conclusions of this manuscript will be made available by the authors, without undue reservation, to any qualified researcher.

## Ethics Statement

This study was carried out in accordance with the recommendations of prenatal diagnosis of Zhejiang Province, prenatal diagnosis center. The protocol was approved by The Ethics Committee for Reproductive Medicine of Taizhou Hospital. All subjects gave written informed consent in accordance with the Declaration of Helsinki.

## Author Contributions

YZ designed the research and wrote the manuscript. QS and JZ performed in the clinic, collected dates, and reviewed the manuscript. QC conducted the statistical analysis. HY, JZ, and XD performed the genetic assays. FJ supervised the research and revised the manuscript.

## Conflict of Interest Statement

The authors declare that the research was conducted in the absence of any commercial or financial relationships that could be construed as a potential conflict of interest. The reviewer QY declared a shared affiliation, with no collaboration, with several of the authors, YZ, QS, QC, HY, and FJ, to the handling Editor at the time of review.

## Footnotes

1 http://dgv.tcag.ca/dgv/app/home

2 http://genome.ucsc.edu

3 www.iscaconsortium.org

4 http://decipher.sanger.ac.uk/patient/
