# Supplementary figures and images for: Comparison of Efficiencies of Non-invasive Prenatal Testing, Karyotyping, and Chromosomal Micro-Array for Diagnosing Fetal Chromosomal Anomalies in the Second and Third Trimesters
Source: Front Genet. 2019 Mar 11;10:69. doi: 10.3389/fgene.2019.00069 (PMC6421281; doi:10.3389/fgene.2019.00069)

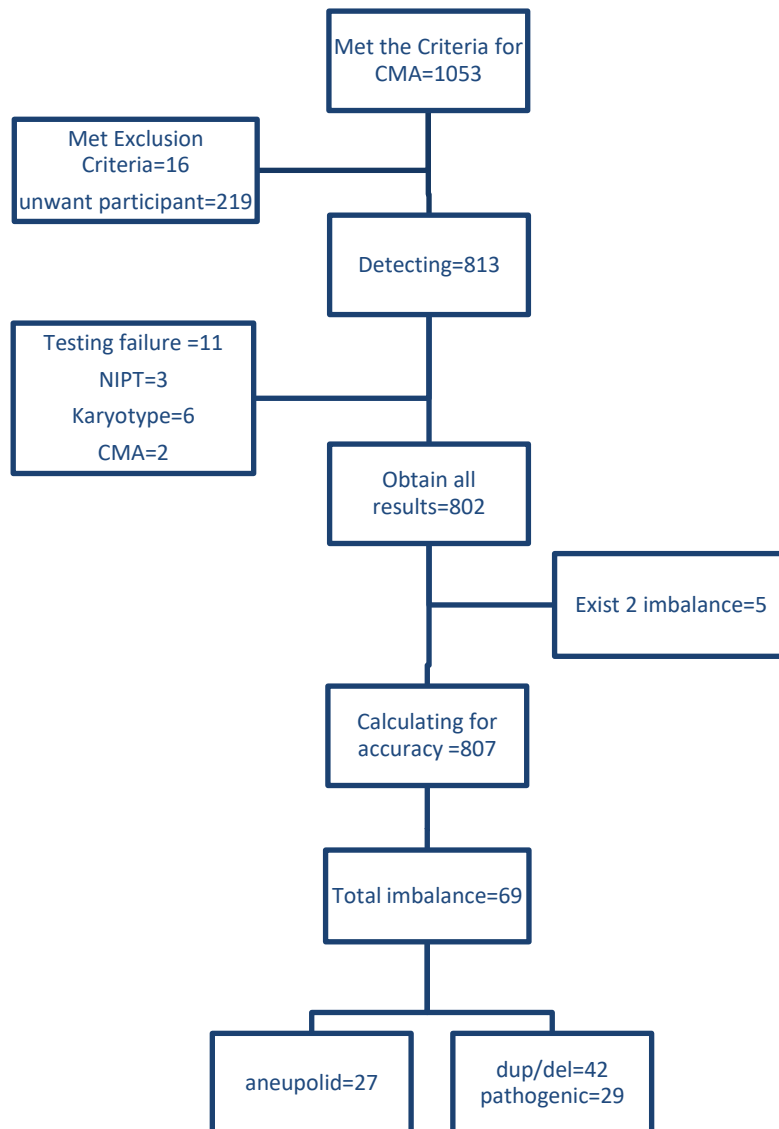

Supplement: Supplementary file 1 [file Image_1.PDF]
