# Supplementary material for: Comparison of Efficiencies of Non-invasive Prenatal Testing, Karyotyping, and Chromosomal Micro-Array for Diagnosing Fetal Chromosomal Anomalies in the Second and Third Trimesters
Source: Front Genet. 2019 Mar 11;10:69. doi: 10.3389/fgene.2019.00069 (PMC6421281; doi:10.3389/fgene.2019.00069)
Supplement: Supplementary file 2 [file Table_1.DOCX]

[Supplementary materials](http://review.frontiersin.org/Document/DownloadSupplementaryMaterial?articleId=414697&userId=565144&roleId=17): Table 1. List of pathogenic CNVs with sizes smaller than 2Mb (only detected by CMA).

| pathogenic or likely dup/del | CNVs-size |
| --- | --- |
| 16p11.2（29,580，020-30,176,508）*1 | 0.596 |
| 16p13.11(14,893,698-16,458,424)*1 | 1.600 |
| Xp22.31（6,724,784-7,827,439）*1 | 1.100 |
| 16p11.2（29,580,020-30,330,881）*3 | 0.715 |
| 16p11.2（29,591,326-30,243,606）*3 | 0.652 |
| 8p23.1（11,290,914-11,935,465）*3 | 0.645 |
| 1q21.1（145,382,123-145,888,926）*1 | 0.507 |
| 22q11.21(18,631,364-19,025,845)*1 | 0.394 |
| 16p11.2(28,708,186-29,051,191)*1 | 0.343 |
| 15q11.2（22,770,421-23,082,237）*1 | 0.312 |
| 17q21.32（45,354,723-45,555,062）*1 | 0.200 |
| 2p16.3（51,015,693-51,164,931）*1 | 0.149 |
| 14q32.32(103,331,589-103,469,914)*1 | 0.138 |
| 6p25.1(5,335,471-5,443,538)*1 | 0.108 |
| 14q21.1(39,553,049-39,615,837)*1 | 0.063 |
